# Pan‐Neurofascin Antibody‐Associated Nodopathy: A Critical Guillain–Barré Syndrome Mimic in the Differential Diagnosis—Report of Two Cases

**DOI:** 10.1155/crcc/2662185

**Published:** 2026-09-18

**Authors:** Andrea Nemethova, Willem De Ridder, Ingrid Baar, Alicia Alonso-Jimenez

**Affiliations:** ^1^ Department of Neurology, Neuromuscular Reference Centre, Antwerp University Hospital, Antwerp, Belgium, uza.be; ^2^ Translational Neurosciences and Peripheral Neuropathy Group, University of Antwerp, Antwerp, Belgium, uantwerpen.be; ^3^ Laboratory of Neuromuscular Pathology, Institute Born-Bunge, University of Antwerp, Antwerp, Belgium, uantwerpen.be; ^4^ Intense Care Unit, Antwerp University Hospital, Antwerp, Belgium, uza.be

**Keywords:** antibody-associated nodo-paranodopathy, autoimmune nodopathy, GBS-mimic, Guillain–Barré syndrome, intensive care, intravenous immunoglobulins, pan-neurofascin, pan-neurofascin antibody-positive autoimmune neuropathy, rituximab, tetraplegia

## Abstract

Guillain‐Barré syndrome (GBS) is an acute autoimmune polyradiculoneuropathy typically characterized by an ascending sensorimotor deficit with potential involvement of bulbar and respiratory muscles that may necessitate intensive care admission and mechanical ventilation. Standard treatment includes supportive care, management of complications such as weakness, immobility, respiratory failure, autonomic dysfunction and pain, along with early initiation of immunotherapy—either intravenous immunoglobulin (IVIg) or plasma exchange (PE). However, lack of significant clinical improvement following immunotherapy should prompt consideration of alternative diagnoses, including pan‐neurofascin antibody‐positive autoimmune nodopathy (panNF + AN). In this report, we present two patients presenting with a severe GBS‐like neuropathy. Initial treatment with IVIg resulted in either no response or only transient, mild improvement, followed by rapid clinical deterioration to near‐complete tetraplegia, respiratory failure, and autonomic and cranial nerve involvement. Both patients were unresponsive to further treatment with a second course of IVIg, PE and corticosteroids. Subsequent diagnostic evaluation revealed the presence of pan‐neurofascin antibodies, confirming panNF + AN. This prompted initiation of treatment with rituximab, which resulted in sustained and complete clinical recovery in both cases. A transient or mild clinical response—or an initial lack of response—to standard GBS‐treatment followed by rapid and severe deterioration in cases initially presenting as GBS should be considered a red flag warranting evaluation for AN‐associated antibodies. Recognition of this important GBS mimic is critical, as it requires an alternative treatment approach with rituximab, which has been associated excellent clinical outcomes.

## 1. Introduction

Guillain–Barré syndrome (GBS) is an acute, autoimmune polyradiculoneuropathy, with classical clinical presentation of progressive sensorimotor deficit in the extremities in combination with decreased or absent tendon reflexes [1]. In addition to limb paresis, patients with GBS can develop weakness of bulbar and respiratory musculature requiring admission to an intensive care unit (ICU) and mechanical ventilation (MV) in up to 30% of the patients [2, 3]. The mortality of this subgroup of GBS patients necessitating MV ranges between 8.3%–20% [4, 5].

Treatment of GBS is supportive medical care (monitoring for bulbar, respiratory and autonomic deterioration), management of complications related to (bulbar) weakness, immobility, respiratory insufficiency, autonomic dysfunction and pain and early start of immunotherapy (intravenous immunoglobulins [IVIg] or plasma exchange [PE]) [6]. Although the response of GBS to immunotherapy is influenced by the underlying type of nerve injury (axonal versus demyelinating), cases that are resistant or refractory to treatment should prompt consideration of pan‐neurofascin‐positive (panNF +) autoimmune nodopathy (AN) [7–15]. These pan‐neurofascin antibodies are directed against an epitope shared by neurofascin (NF)‐140/186 (which attaches the axolemma to the extracellular matrix at the node of Ranvier) and NF‐155 (which attaches the myelin to contactin‐1 and contactin‐associated protein‐1 of the axon at the paranode) [16].

In this article, we describe two patients presenting with acute, severe GBS‐like neuropathy with tetraplegia and of respiratory failure, refractory to treatment of IVIg, in whom establishing the correct diagnosis enabled appropriate therapy and resulted in a favourable clinical outcome.

## 2. Presentation of Cases

### 2.1. Patient 1

**Figure 1:**
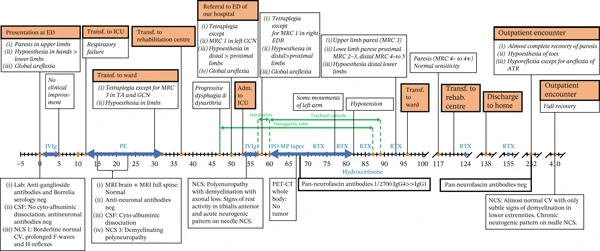
Clinical course of Patient 1. For details, see main text. ED, emergency department; ICU, intensive care unit; MRC, Medical Research Council paresis scale (maximum 5); CSF, cerebrospinal fluid; NCS, nerve conduction study; CV, conduction velocity; MRI, magnetic resonance imaging; PET‐CT, positron emission tomography‐computed tomography; IVIg, intravenous immunoglobulin; PE, plasma exchange; PO MP, oral methylprednisolone; RTX, rituximab; Transf., transfer; Adm., admission; Rehab., rehabilitation; Neg, negative; Pos, positive; TA, tibialis anterior; GCN, gastrocnemius; ATR, Achilles tendon reflexes.

A 73‐year‐old man, without relevant medical history, developed progressive paraesthesia in the hands and ascending paraesthesia in the lower extremities over 5 days followed by distal weakness in the four limbs. He subsequently presented at the emergency department of a hospital (Day 0, Figure 1). On neurologic examination at admission, the patient showed distal‐predominant weakness of the upper limbs, hypoesthesia in the hands and lower extremities, generalized areflexia and gait instability. Antiganglioside antibodies and Borrelia serology were negative. Cerebrospinal fluid (CSF) analysis was normal. Nerve conduction studies (NCS) showed borderline normal conduction velocities (CV) and prolonged F‐waves and H‐reflexes. Following the tentative diagnosis of GBS, the patient was treated with IVIg (2 g/kg over 5 days) without clinical improvement.

On Day 12, there was a clinical deterioration with development of respiratory failure leading to transfer from the ward to ICU for PE (1 session every 2 days for 12 days). MRI of the brain and spinal cord was normal. Repeat CSF analysis on Day 21 showed elevated protein levels (103 mg/dL) and no pleocytosis, compatible with albuminocytologic dissociation, a typical finding in GBS. NCS and EMG on Day 23 showed demyelinating polyneuropathy, compatible with GBS.

After completion of PE, the patient was retransferred to the ward. Clinical neurologic examination revealed near‐complete tetraplegia, with residual strength (Medical Research Council [MRC] 3/5) only in tibialis anterior and gastrocnemius muscles, accompanied by hypoesthesia in the extremities. On Day 37, the patient was transferred to a rehabilitation centre.

**Figure 2:**
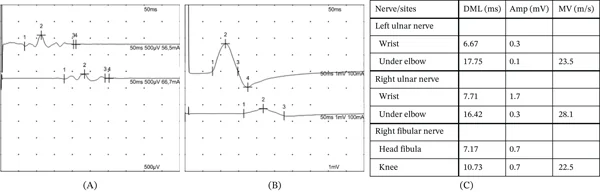
Motor conduction studies (Case 1) of (A) left ulnar nerve and (B) right ulnar nerve derived from abductor digiti minimi, stimulated at the wrist (top curve) and under the elbow (bottom curve). Note the (A) temporal dispersion and (B) conduction block. (C) Distal motor latency (DML), compound motor action potential (CMAP) amplitudes (Amp) and motor velocity (MV) of the ulnar nerves and right fibular nerve. Note the greatly reduced CMAP amplitudes, greatly prolonged distal motor latencies and significantly reduced motor velocities (below 30 m/s).

On Day 47, the patient developed aspiration pneumonia due to progressive dysphagia and dysarthria and was referred to the emergency department of our hospital 3 days later. Clinical examination revealed complete tetraplegia, hypoesthesia in the extremities, with striking loss of proprioception (profound loss of position sense in the upper extremities) and global areflexia. The patient was diagnosed with hospital‐acquired pneumonia and was admitted to the ICU for respiratory monitoring and noninvasive ventilation. The atypical clinical course, with minimal response to initial IVIg and subsequent sudden deterioration, prompted consideration of alternative diagnoses. NCS and electromyography (EMG) demonstrated evidence of demyelination and axonal loss (Figure 2), with active denervation and an acute neurogenic pattern observed on needle EMG. Whole body positron emission tomography‐computed tomography (PET‐CT) revealed no tumours. Serum (para)nodal antibody testing only returned on Day 68, demonstrating positivity for pan‐NF antibodies (titre 1:2700, IgG4> > IgG1), confirming the diagnosis of panNF + AN.

In the time leading up to the eventual diagnosis, a second course of IVIg (2 g/kg over 5 days) was administered starting on Day 53, followed by methylprednisolone (MP) taper beginning on Day 60, which resulted in no clinical improvement. Moreover, on Day 57 (i.e., 7 days after admission to the ICU), the respiratory function of the patient further deteriorated, necessitating intubation and invasive ventilation. On Day 60, a tracheostomy was performed.

Upon confirmation of the diagnosis of panNF + AN, treatment with rituximab (RTX) (375 mg/m^2^ body surface area) was started on Day 72, consisting of four weekly infusions followed by two monthly infusions. The first improvement in extremity strength (i.e., some movements in left arm) was observed in Day 77 (5 days after initiation of RTX). On Day 82, when oral MP was discontinued, autonomic dysfunction (hypotension) was observed, which was treated with stress‐dose of hydrocortisone.

In response to the established therapy regimen, the patient progressively improved. Dysphagia resolved (allowing removal of the nasogastric tube on Day 86), respiratory function recovered (decannulation was performed on Day 89). Limb weakness gradually improved: on Day 93 (the day of the fourth RTX infusion), strength in the upper extremities was MRC 4 proximally and MRC 3 distally, whereas strength in the lower extremities was MRC 2–3 proximally and MRC 4–5 distally. Sensory deficits also improved, with only distal lower extremity hypoesthesia remaining on Day 93. On Day 96, the patient was transferred to the neurology ward, where further improvement was observed. By the time of discharge to the rehabilitation centre, limb strength had further improved to MRC 4 and sensation in the limbs had returned to normal, except for the toes.

First outpatient encounter was on Day 232 when the weakness had almost completely recovered (except for MRC 4 in tibialis anterior and abductor pollicis brevis) with hypoesthesia limited to the toes and partial recovery of the tendon reflexes. Pan‐NF antibodies were negative. Second outpatient encounter on Day 410 showed full recovery. NCS and EMG showed near normalisation of CV with only subtle signs of demyelination in the lower extremities and no signs of active denervation.

### 2.2. Patient 2

**Figure 3:**
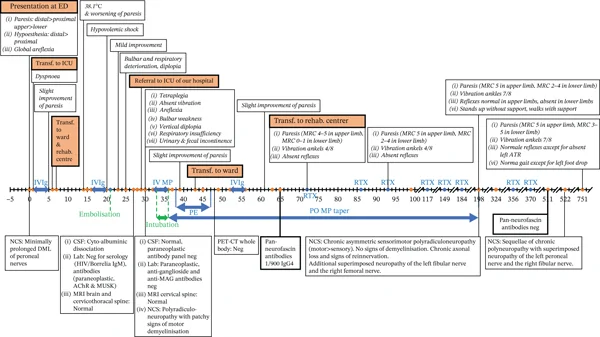
Clinical course of Patient 2. For details, see main text. ED, emergency department; ICU, intensive care unit; MRC, Medical Research Council paresis scale (maximum 5); CSF, cerebrospinal fluid; DML, distal motor latency; CV, conduction velocity; CMAP, compound motor action potential; CXR, chest x‐ray; CRP, C‐reactive protein; Hb, haemoglobin; MRI, magnetic resonance imaging; PET‐CT, positron emission tomography‐computed tomography; IVIg, intravenous immunoglobulin; PE, plasma exchange; IV MP, intravenous methylprednisolone; PO MP, oral methylprednisolone; RTX, rituximab; Transf., transfer; Adm., admission; Rehab., rehabilitation; Neg, negative; Pos, positive; ATR, Achilles tendon reflexes; NCS, nerve conduction study.

A 70‐year‐old man, with a 15‐year prior history of myocardial infarction, a 6‐years prior history of cardiac ventricular thrombus and a 3‐years history of prostate cancer, presented to the emergency department (Day 0, Figure 3) with sensory disturbances in his extremities that had developed over the preceding 5 days. Clinical examination revealed limb weakness, more pronounced distally and in the upper limbs, distal‐predominant hypoesthesia in the, global areflexia and gait disturbance. NCS showed minimally prolonged distal motor latency (DML) of the peroneal nerves. A lumbar puncture was not performed due to ongoing anticoagulant therapy for his cardiac history. Since the clinical presentation was suggestive of GBS, treatment with IVIg (same regimen as in Case 1) was started. The day after initial presentation, the patient developed dyspnoea and was transferred to the ICU. On the day of completion of the treatment with IVIg (Day 5), he showed some clinical improvement and was allowing transfer first to the ward (Day 6) and then subsequently to the rehabilitation centre (day 7).

Nine days after completion of the treatment with IVIg (Day 14), the patient developed a subfebrile temperature (38.1°C) and the paresis worsened. As the paresis kept worsening, even when the work‐up for infection turned out negative and the temperature spontaneously normalised, a second round of IVIg was administered (on Days 16–20).

On Day 20, the patient became hypotensive, tachycardic and progressively anaemic, subsequently developing acute renal and hepatic failure. The cause of this hypovolemic shock was identified as bleeding from a lumbar branch of the aorta. This complication was successfully treated with embolization of the affected lumbar branch and drainage of the retroperitoneal hematoma.

Neurological evaluation following the second course of IVIg could only be performed on Day 23 due to this complication and revealed no clinical improvement. Moreover, over the following 2 days, the patient developed additional progressive dysarthria, hypophonia, dysphagia, dyspnoea and intermittent vertical diplopia. A lumbar puncture was performed on Day 27 following temporary anticoagulation suspension for the bleeding complication, demonstrating albuminocytologic dissociation. Laboratory studies, including HIV serology, Borrelia IgM, paraneoplastic, acetylcholine receptor (AChR) and muscle‐specific kinase (MUSK) antibodies, were all negative. Brain and cervicothoracic spine MRI findings were normal. Repeat NCS on Day 28 demonstrated prolonged DMLs and decreased compound motor action potential (CMAP) amplitudes in both upper and lower extremities. Upper extremity CV remained normal, whereas EMG revealed evidence of active denervation and accelerated contraction.

Because of clinical deterioration despite a second course of IVIg, the patient was referred to the ICU of our hospital (Day 29). Clinical examination revealed tetraparesis, sparing only MRC 1–2 in the distal lower extremities, absent vibration sense and tendon reflexes in all extremities, bifacial weakness, dysarthria, hypophonia, intermittent vertical diplopia, respiratory insufficiency and urinary and faecal incontinence. Repeat CSF, including paraneoplastic antibody panel, was negative. Blood analysis for paraneoplastic, antiganglioside and antimyelin associated glycoprotein (MAG) antibodies was negative as well. NCS and EMG showed a polyradiculoneuropathy with patchy signs of motor demyelination.

The patient′s clinical course thus far has deviated from the typical presentation of GBS, as a clear nadir was not reached and the patient showed a fluctuating course without meaningful improvement after IVIg. Therefore, alternative diagnoses were considered.

First, acute‐onset chronic inflammatory demyelinating polyradiculoneuropathy (CIDP) was suspected, and a treatment trial with IV MP and PE was initiated. Almost simultaneously with the treatment with IV MP, due to respiratory failure, the patient required intubation for 4 days. Following PE, there was only slight improvement in muscle strength, with MRC‐scores 0–2 proximally and MRC 5‐ distal in the upper limbs, and MRC 0–1 proximally and MRC 1–4 distally in the lower limbs, as well as mild improvement in vibration sense.

Secondly, tumoral involvement was assessed by whole‐body PET‐CT, which demonstrated no abnormal findings.

Third, AN was considered, prompting testing for nodal and paranodal antibodies. While awaiting the results of these antibodies, a third treatment with IVIg was initiated on Day 52, resulting in only minimal improvement of lower limb paresis on Day 61. On Day 65, testing returned positive for pan‐NF antibodies (titre 1:900, IgG4), confirming the diagnosis of a AN. Consequently, treatment with RTX was initiated on Day 72 using the same regimen as in Patient 1. As a result of this treatment, there was a gradual significant improvement of the clinic, with full recovery of the paresis in the upper extremities on clinical examination 2 weeks after the first treatment. Strength of lower extremities recovered almost entirely, as evaluated clinically on last outpatient visit approximately 1 year after the last RTX administration (Day 751). The residual paresis of the right hip flexion and left drop foot was attributed to a superimposed neuropathy of, respectively, the right femoral and left fibular nerves, as confirmed by follow‐up EMGs. These EMGs showed progressive recovery of previous signs of demyelination and axonal loss.

## 3. Discussion

We report two cases of panNF + AN that initially presented as GBS mimics. These observations are consistent with previously published reports describing 14 patients (Table S1, Supporting Information) [7–15]. Recognition of panNF + AN as a rare GBS mimic is critical, given its markedly different prognostic and therapeutic implications. However, early clinical and paraclinical assessments are often misleading, as there are no definitive initial features suggesting panNF + AN.

In both patients, initial neurological examination demonstrated weakness predominantly affecting upper limbs and distal segments, distal‐predominant hypoesthesia, generalized areflexia and gait disturbance. In contrast to the distal predominance of motor involvement observed in our cases, most reported cases of panNF + AN with a GBS‐like presentation showed predominantly proximal weakness [7, 12, 14]. By comparison, the distal predominance of sensory disturbances seen in both of our patients was consistent with all six‐reported panNF + AN cases with a GBS‐like presentation in which sensory involvement was described [7, 8, 11, 13–15].

Although albuminocytologic dissociation is a recognized hallmark of GBS, it typically emerges only in the later stages of the disease, as observed in our two patients on Days 21 and 27, respectively, thereby limiting its utility for early diagnosis. Similarly, albuminocytologic dissociation in CSF has been reported in 5 of 8 panNF + AN cases with a GBS‐like presentation for which data were available [10, 11, 13–15].

Serial NCS in our two patients were initially interpreted as normal but subsequently demonstrated features of both demyelination and axonal involvement. In the literature, panNF + AN cases with a GBS‐like presentation show either demyelinating or mixed demyelinating–axonal patterns on NCS in similar proportions (4 of 8 reported cases) [7–11, 15]. The conventional dichotomy classifying acute poly (radiculo) neuropathies as either demyelinating or axonal can be misleading. A reduced CMAP amplitude does not necessarily indicate irreversible axonal loss; it may instead reflect reversible conduction blocks due to demyelination). Therefore, decreased CMAP amplitudes can be misinterpreted either as distal axonal degeneration or a demyelinating conduction block. In some cases, a reduced CMAP amplitude may even represent transient, reversible dysfunction of the axolemma rather than true axonal degeneration [2].

The diagnosis of panNF + AN in both patients was established based on the detection of pan‐NF antibodies with IgG4 subclass predominance. Although antibody titres are considered markers of disease severity and are sensitive to relapses, they do not predict prognosis but remain useful for monitoring disease activity [15]. The IgG subclass of the pan‐NF antibodies is of great importance for the treatment. IgG4 predominance renders the panNF + AN patient irresponsive to treatment with IVIg, as opposed to IgG1‐3 subclasses [17]. In contrast to IVIg, RTX has been described as an effective treatment of IgG4 neurological disorders [17, 18].

It is important to emphasise that assays for these antibodies remain of limited availability. When accessible and performed according to quality standards, they should be used in all patients with GBS or CIDP, as recommended by the EAN/PNS guidelines [19]. However, as testing is unavailable in most centres, patients should be selected based on specific clinical features. Our cases highlight two clinical ‘red flags’ that should prompt consideration of this diagnosis. First, a lack of significant response (Case 1), only a transient minimal response (Case 2), or further clinical deterioration (both cases) following IVIg and PE—therapies generally effective in GBS—should prompt clinicians to suspect the GBS mimic panNF + AN. Consistent with our findings, among 13 reported panNF + AN cases with a GBS‐like presentation treated initially with IVIg, 6 showed no clinical improvement and 7 only transient improvement, with all but one of the latter subsequently deteriorating to tetraplegia [7, 9–15]. In Case 2, the transient responses observed after two courses of IVIg were initially interpreted as treatment‐related fluctuations, defined as clinical deterioration within 2 months of GBS onset following prior stabilization or therapeutic improvement [20]. However, the subsequent occurrence of a fulminant relapse after initial improvement is not consistent with treatment‐related fluctuations, and necessitated a reassessment of the original diagnosis.

Second, pronounced sensory loss in the limbs should be regarded as another ‘red flag’ for panNF + AN. In Case 1, the patient exhibited complete loss of proprioception, whereas in Case 2, vibration sense was entirely absent. This distinctive feature may have been overlooked in previously published cases, but due to its clear and unique nature, we regard it as an important red flag.

In summary, panNF + AN represents a GBS mimic with a potentially fulminant course, which may result in tetraplegia, respiratory failure, cranial nerve involvement, autonomic dysfunction and death. Resistance to, or only transient and partial improvement after, standard GBS therapy with IVIg, together with marked sensory involvement (i.e., complete loss of proprioception), should be regarded as key ‘red flags’ prompting consideration of panNF + AN. Close collaboration between intensivists and neurologists is key to early identification of panNF + AN, as prompt treatment with RTX, rather than standard GBS therapy, has been associated with high rates of complete recovery in most reported cases.

## Funding

No funding was received for this manuscript.

## Consent

No written consent has been obtained from the patients.

## Conflicts of Interest

The authors declare no conflicts of interest.

## Supporting information


**Supporting Information** Additional supporting information can be found online in the Supporting Information section. Table S1: Overview adult cases and case series of patients with GBS‐like presentation which eventually received diagnosis of PanNF + AN.

## Data Availability

The data that support the findings of this study are available from the corresponding author upon reasonable request.
