# Supplementary material for: Pan‐Neurofascin Antibody‐Associated Nodopathy: A Critical Guillain–Barré Syndrome Mimic in the Differential Diagnosis—Report of Two Cases
Source: Case Rep Crit Care. 2026 Sep 18;2026:2662185. doi: 10.1155/crcc/2662185 (PMC13589164; doi:10.1155/crcc/2662185)
Supplement: Supplementary file 1 [file CRCC-2026-2662185-s001.docx]

**Supplementary Material**

|  | Gender, age (y) | Preceded by infection/vaccination | Concomitant disease | Clinical manifestation |  |  |  | Timing presentation in relation to onset symptoms  Initial diagnosis | Albuminocytoloci dissociation in CSF | Nerve conduction studies | Pan-neurofascin antibody titre  Isoform | Treatment and response |
| --- | --- | --- | --- | --- | --- | --- | --- | --- | --- | --- | --- | --- |
|  |  |  |  | Sensorimotor distribution | Cranial nerve involvement | ICU admission  Mechanical ventilation | Autonomic involvement |  |  |  |  |  |
| Vallat et al, 2018 | M: in 70’s | No | Low-grade IgA-lambda myeloma | Sensory (distal)  Motor (proximal predominance) | Yes | Yes  Yes | Not available | 8 days  GBS-like | Not available | Demyelinating | 1:8000 (at nadir)  1:200 (at 8 months)  Undetectable (at 9 months)  IgG3 | - IVIg: transient improvement, followed by deterioration to tetraplegia 3 weeks later  - 2nd IVIg: no effect  - PE: no effect  - Steroids: no effect  - 9 months after onset: spontaneous full recovery of motor symptoms, only paraesthesia of lower limb |
| Stengel et al, 2019 | F, 74 | Respiratory infection | Diabetes mellitus | Sensory (distal)  Motor (almost tetraplegia over course of few days) | Yes | Yes  No | Yes | GBS | No | Demyelinating>axonal | NF186: 1:200  NF155: 1:300  Weak IgG3 | - PE: complete recovery |
| Fehmi et al, 2021  n = 8 | ? | Not stated | Nephrotic syndrome | Sensory  Motor | Yes | Yes  Yes | Yes | GBS | ** | Absent CMAP/SNAP responses | NF185: 1:3200  NF155: 1:6400  IgG1 | - IVIg: no effect  - PE: no effect  - Death due to withdrawal of ventilatory support as treatment was ineffective |
|  | ? | Not stated | No | Sensory  Motor | Yes | Yes  Yes | Yes | GBS | ** | Demyelinating | NF185: 1:800  NF155: 1:400  IgG1 | - IVIg: transient/partial effect  - PE: transient/partial effect  - Steroids: transient/partial effect  - RTX: full recovery 9 months post RTX |
|  | ? | Not stated | Nephrotic syndrome | Sensory  Motor | Yes | Yes  Yes | Yes | GBS | ** | Mixed | NF185: 1:6400  NF155: 1:3200  IgG1 | - IVIg: no effect  - PE: no effect  - Steroids: transient/partial effect  - RTX: clinical improvement to MRS 1 18 months post RTX |
|  | ? | Not stated | No | Sensory  Motor | Yes | Yes  Yes | Yes | GBS | ** | Not available | NF185: 1:800  NF155: 1:800  IgG1 | - IVIg: no effect  - Death due to withdrawal of ventilatory support as treatment was ineffective |
|  | ? | Not stated | Nephrotic syndrome | Motor | Yes | No  No (deemed unsuitable) | Yes | GBS | ** | Not available | NF185: 1:400  NF155: 1:400  IgG1 | - IVIg: no effect  - Death because mechanical ventilation deemed unsuitable during the covid pandemic |
| Scheibe et al, 2022 | M, 52 | Vaccination against tetanus/poliomyelitis/diphtheria | Smouldering multiple myeloma | Sensory  Motor (tetraplegia) | Yes | Yes  Yes | Not available | GBS | Yes | Fulfilment of criteria for CIDP | NF186: 1:100  NF155: 1:10  Not stated | - IVIg: transient followed by deterioration to tetraplegia 1 month later  - PE: transient improvement  - Steroids: transient improvement  - RTX: transient improvement  - Daratumumab + lenalidomide + steroids: complete resolution except for a minor paresis of his right big toe and hypesthesia on the backs of his feet |
| Wang et al, 2022 | M, 39 | Not stated | Not stated | Sensory (initially distal, later entire limbs)  Motor (lower limbs) | No | No  No | No | GBS (initially), CIDP (later) | Yes | Mainly demyelinating | NF186: 1:100  NF155: 1:1000  IgG4 | - IVIg: initially not administered, 2 months later no effect  - Steroids: improvement of sensory symptoms  - CY: no effect  - RTX: ability to walk 100m, improvement of distal limb paraesthesia |
| Acerra et al, 2024 | F, 77 | No | Not stated | Sensory  Motor (initially proximal, later tetraplegia) | Yes | Yes  Yes | Not stated | GBS | No | Demyelinating | “High levels”  Not stated | - IVIg + steroids: transient improvement followed by dramatic deterioration 3 weeks later, died due to septic complications |
| Krim et al, 2025 | M, 52 | No | Not stated | Sensory (distal)  Motor (initially distal, later tetraplegia) | Complete CN dysfunction, locked-in | Yes  Yes | Yes | GBS | Yes | Demyelinating | Titre not mentioned  IgG4 | - IVIg: no effect  - PE: no effect  - RTX: gradual clinical improvement |
| Kmezic et al, 2025  (Swedish) | M, 57 | Not stated | Not stated | Sensory  Motor (proximal predominance) | Yes | Yes  Yes | Yes | 5 days  GBS | Yes | Demyelinating | 1:500  IgG4 | - IVIg: transient improvement, followed by deterioration to tetraplegia  - Steroids: no effect  - RTX: good recovery with residual distal weakness |
|  | F, 49 | Not stated | Not stated | Sensory (distal)  Motor (proximal predominance) | No | Yes  Yes | Yes | 3 days  GBS | No | Demyelinating | 1:200  IgG4 | - IVIg: transient improvement, followed by deterioration to tetraparesis  - 2nd IVIg: initially stabilisation, followed by further deterioration to tetraplegia  - RTX: almost complete recovery (slight weakness in hands) |
| Cabral et al, 2025 | F, 84 | No | Non-metastatic breast cancer | Motor  Sensory (distal predominance) | Yes | No  No | Yes | 1 week  GBS | Yes | Initially axonal, later mixed | 1:900  IgG4 | - IVIg: transient improvement followed by deterioration to tetraparesis  - 2nd IVIg: no effect  - Steroids: no effect  - RTX: almost complete recovery (slight ataxic gait) (clinical improvement observed before start of RTX) |
| Nemethova et al, 2025 | M, 73 | No | No | Sensory (distal predominance)  Motor (initially only in upper limb and distal predominance, later tetraplegia) | No | Yes  Yes | Yes | 5 days  GBS | Yes | Initially normal, later demyelinating>axonal | 1:2700 (IgG4>>IgG1) | - IVIg: transient improvement, followed by deterioration to tetraplegia  - PE: no effect  - 2nd IVIg + steroids: no effect  - RTX: full recovery |
|  | M, 70 | No | Psoas haemorrhage | Sensory (distal predominance)  Motor (inititally distal predominance, later tetraplegia) | Yes | Yes  Yes | Yes | 5 days  GBS | Yes | Initially normal, later demyelinating>axonal | 1/900  IgG4 | - IVIg: transient improvement followed by worsening of paresis  - 2nd IVIg: conflicting data: Mild clinical improvement (in records) vs no clinical improvement (according to patient) followed by tetraplegia, dyspnoea and cranial nerve involvement  - Steroid + PE: slight improvement of paresis and vibration sense  - 3rd IVIg: slight improvement of paresis  - RTX: full recovery except for residual paresis of the right hip flexion and left drop foot (attributed to a superimposed neuropathy) |

**Table S1**: Overview adult cases and case series of patients with GBS-like presentation which eventually received diagnosis of autoimmune nodo-paranodopathy with pan-neurofascin antibodies. ICU = intensive care unit. CSF = cerebrospinal fluid. IVIg = intravenous immunoglobulin. PE = plasma exchange. CY = cyclophosphamide. RTX = rituximab.

** no individual data available (ranging from no or mild cyto-albumin dissociation, evaluated in 7 out of 8 patients (these patients include those with GBS-like presentation and those with CIDP-like presentation))
